# Mapping internal brainstem structures using MP2RAGE derived T1 weighted and T1 relaxation images at 3 and 7 T

**DOI:** 10.1002/hbm.24938

**Published:** 2020-01-23

**Authors:** Susanne G. Mueller

**Affiliations:** ^1^ Department of Radiology University of California San Francisco California

**Keywords:** 3 T, 7 T, brainstem, MP2RAGE, nuclei, segmentation, tract

## Abstract

The brainstem is a site of early pathology in several neurodegenerative diseases. The overall goal of this project was (a) To develop a method to segment internal brainstem structures from MP2RAGE derived images. (b) To compare the segmentations at 3 and 7 T. (c) To investigate age effects on intensities and segmentations. MP2RAGE derived T1 weighted images (UNI) and T1 relaxation maps (T1map) were obtained from two public data sets (LEMON: 50 3 T data sets, ATAG: 46 7 T data sets). The UNI and T1map images were rescaled using a linear scaling procedure and a ratio (RATIO) image calculated. The brainstem was extracted and k‐mean clustering used to identify six intensity clusters from the UNI, T1map and RATIO at 3 and 7 T. Nonlinear diffeomorphic mapping was used to warp the six intensity clusters in subject space into a common space to generate probabilistic group averages/priors that were used to inform the final probabilistic segmentations at the single subject level for each field strength. The six clusters corresponded to six brainstem tissue types (three gray matter clusters and two white matter clusters and one csf/tissue boundary cluster). The quantitative comparison of the 3 and 7 T probabilistic averages showed subtle differences that affected the localization of age‐associated brainstem volume losses. The segmentation approach presented here identified the same brainstem gray and white matter structures at both field strengths. Further studies are necessary to investigate how resolution and field strength contribute to the subtle differences observed at the two field strengths.

## 1 INTRODUCTION

The brainstem is a complex structure consisting of densely packed and often not well‐delineated fiber tracts and small nuclei that play a crucial role in a variety of functions including locomotion, sensory processing, autonomic control, consciousness, and even cognition. Disturbances of brainstem functions are thought to be an early symptom of several neurodegenerative diseases, for example, progressive supranuclear palsy, Alzheimer's and Parkinson's disease, and are also known to contribute to other disabling chronic conditions, for example, chronic pain, stress, sleep disorders, and epilepsy to name just a few (Anaclet & Fuller, 2017; Mills et al., 2018; Mueller et al., 2018; Rüb et al., 2016; Weinshenker, 2018; Withwell et al., 2017). Given the brainstem's role in normal and pathological brain processes there has been a considerable interest in assessing brainstem structure and function in vivo. Except for the substantia nigra and nucleus (ncl.) ruber, brainstem nuclei, and tracts are usually not discernible in MR images acquired for clinical purposes which prompted the development of several dedicated brainstem sequences. These included a three‐dimensional (3D) multi‐echo FLASH sequence for multiparametric mapping of brainstem structures (Helms, Dathe, & Dechent, 2008; Lambert, Lutti, Helms, Frackowiak, & Ashburner, 2014), high resolution DTI and fMRI sequences to depict white matter fiber tracts and functional subdivisions of the periaqueductal gray (Bianciardi et al., 2018; Faull, Jenkinson, Clare, & Pattinson, 2015; Sclocco, Beissner, Bianciardi, Polimeni, & Napadow, 2018) and sequences optimized to detect brainstem structures with high iron or neuromelanin content, for example, quantitative susceptibility imaging to image the substantia nigra and ncl. ruber, or T1‐weighted turbo‐spin echo and magnetization transfer weighted sequences to image the locus coeruleus (Betts, Cardenas‐Blanco, Kanowski, Jessen, & Düzel, 2017; Keuken et al., 2017; Liu et al., 2019; Priovoulos et al., 2018; Sasaki et al., 2006; Straub et al., 2019). Many of these sequences were implemented on 7 T magnets whose higher signal to noise and resolution are better suited to depict small brainstem structures. While these specialized sequences are often able to depict the targeted brainstem structures with impressive detail the need to use dedicated imaging protocols frequently combined with sequences requiring long (>10 min) acquisition times and/or 7 T magnets has prevented these techniques from being implemented into clinical imaging protocols.

The MP2RAGE sequence is a variation of the standard magnetization prepared rapid gradient echo (MPRAGE) sequence. In contrast to the latter, the MP2RAGE acquires two gradient echo images with different inversion times that can be combined 1. To obtain a T1‐weighted image (here referred to as UNI) that is free of proton density and T2* contrast and has a greatly reduced reception bias field and transmit field inhomogeneity and 2. To calculate a high resolution whole brain T1 relaxation map using a look‐up table (here referred to as T1_map; Marques et al., 2010; Marques & Gruetter, 2013). Taken together, the MP2RAGE sequence provides a high resolution T1‐weighted image with excellent cortical and subcortical gray/white contrast and a high resolution T1 relaxation map suitable for myelin mapping (Kim & Knösche, 2016; Lutti, Dick, Sereno, & Weiskopf, 2014; Stueber et al., 2014), that is, crucial information for depicting fiber tracts and small nuclei. The MP2RAGE sequence is part of the latest software releases of 3 and 7 T Siemens magnets and is available as research sequence on Philips magnets. The acquisition times of a high resolution (0.7–1 mm^3^) whole brain image are between 8–12 min. making its implementation into a clinical imaging protocol realistic (e.g., Okubo et al., 2016; Simioni et al., 2014).

The overall goals of this project were therefore (a) to develop a method to segment internal brainstem structures using the MP2RAGE sequence, (b) to compare the segmentation quality at 3 and 7 T, and (c) to investigate age effects on grayscale intensities of internal brainstem structures at 3 and 7 T, their impact on tissue segmentation and the ability to detect age effects with deformation‐based morphometry.

## 2 METHODS

### 2.1 Population

The T1‐weighted images and the T1‐relaxation map images derived from the MP2RAGE sequence from two publicly available data sets were used for this project.

1. Leipzig Study for Mind–Body–Emotion Interactions (LEMON) project. A detailed description of this data set can be found in Babayan et al. (2019). The study population consisted of 227 healthy participants falling into two age groups [20–35 years old: *n* = 153, mean (*SD*) age = 25.1 (3.1) years and 59–77 years old (*n* = 74, mean (*SD*) age = 67.6 (4.7) years]. 50 (m/f: 21/29) subjects reflecting the age distribution observed in the whole population whose images had good quality on visual inspection were randomly selected and divided into two age groups: (a) young (20–35 years of age): *n* = 31 and (b) old (59–77 years of age): *n* = 19.
2. Atlasing of the basal ganglia (ATAG) project. A detailed description of this data set can be found in Forstmann et al. (2014). The study population consisted of young participants [*n* = 30, mean (*SD*) age 23.8 (2.3) years], middle‐aged participants [*n* = 14, mean age (*SD*) 52.5 (6.6) years], and older participants (*n* = 10, mean (*SD*) age 69.6 (4.6) years), 46 (m/f: 23/23) of these 54 data sets were selected based on image quality in lower brain/brainstem regions on visual inspection and divided into two age groups: (a) young (20–45 years of age): *n* = 29 and (b) old (45–75 years of age): *n* = 17.

### 2.2 Imaging protocol

#### 2.2.1 LEMON

All subjects underwent a standardized imaging protocol on the same 3 Tesla scanner (MAGNETOM Verio, Siemens Healthcare GmbH, Erlangen, Germany) equipped with a 32‐channel head coil that included a MP2RAGE with the following parameters: Sagittal orientation, 176 slices, TR = 5,000 ms, TE = 2.92 ms, TI1 = 700 ms, TI2 = 2,500 ms, FA1 = 4°, FA2 = 5°, echo spacing = 6.9 ms, bandwidth = 240 Hz/pixel, FOV = 256 mm, voxel size = 1 × 1 × 1 mm, GRAPPA acceleration factor 3, acquisition time: 8 min 22 s.

#### 2.2.2 Atlasing of the basal ganglia

All subjects underwent a standardized imaging protocol on the same 7 T Siemens Magnetom MRI scanner equipped with a 24‐channel head array Nova coil (NOVA Medical Inc., Wilmington, MA) that included a MP2RAGE with the following parameters: Sagittal orientation, 240 slices, TR = 5,000 ms, TE = 2.45 ms, TI1 = 900 ms, TI2 = 2,750 ms, FA1 = 5°, FA2 = 3°, echo spacing = 6.8 ms, bandwidth = 250 Hz/pixel, FOV = 224 mm, voxel size = 0.7 × 0.7 × 0.7 mm, GRAPPA acceleration factor 2, acquisition time: 10 min 57 s.

### 2.3 Image processing and brainstem segmentation

**Figure 1:**
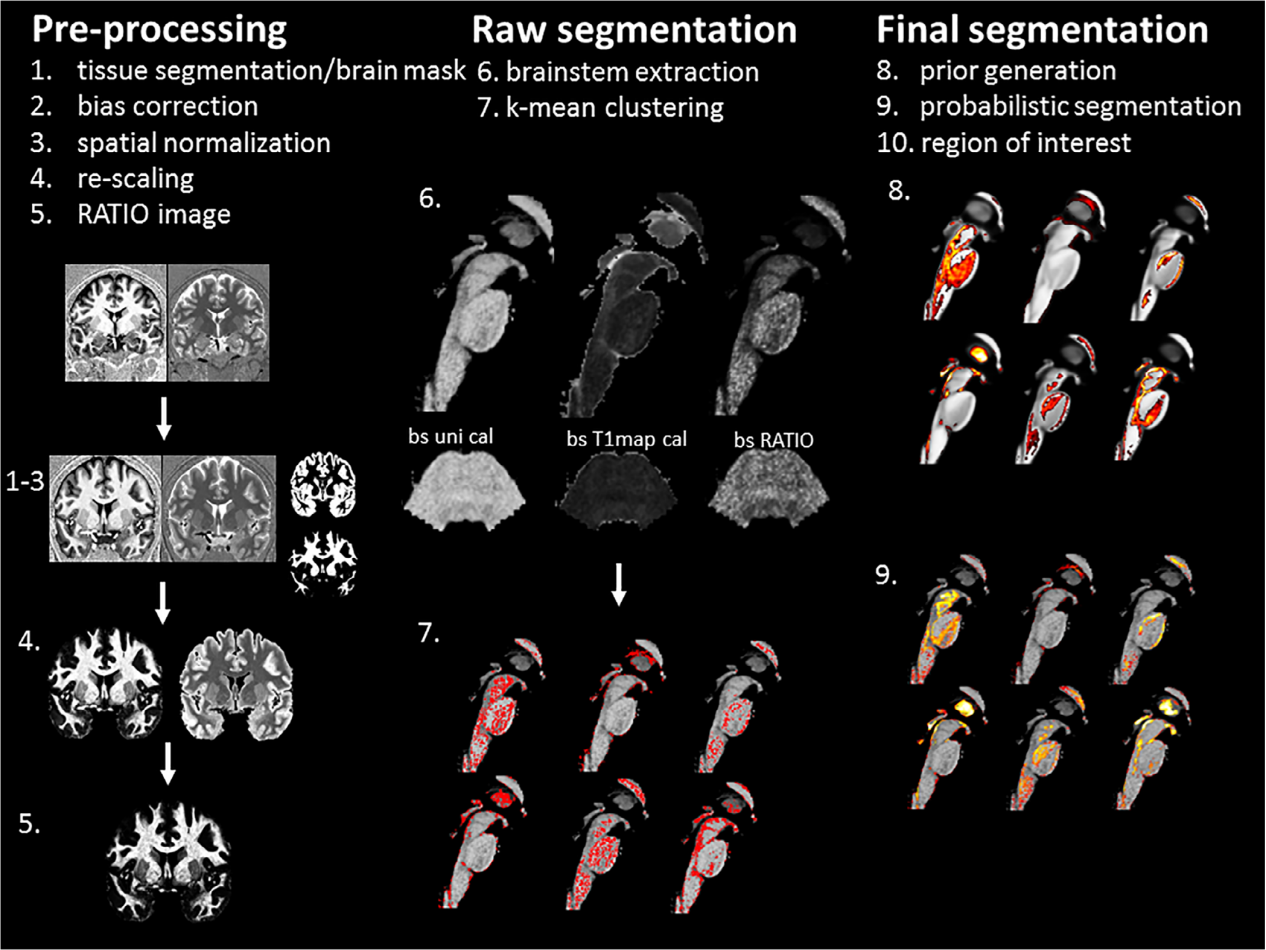
Summary of brainstem segmentation pipeline. The pipeline consists of three main modules of which each encompasses several steps. The first module is “preprocessing” that uses SPM12 routines for tissue segmentation with inbuilt additional bias correction and brainmask generation, followed by spatial normalization to the MNI space while maintaining the original image resolution (Steps 1–3). The resulting outputs (UNI and T1_map) are rescaled (Step 4) generating the outputs, UNI_cal and T1_map_cal that are then used to calculate the RATIO image (Step 5). The rescaled images are passed on to the second module whose first step is to use a binary brainstem/thalamus mask in MNI space to extract the brainstem/diencephalon thereby generating bs_UNI, bs_T1_map, and bs_RATIO (Step 6). Step 7 uses a k‐mean clustering algorithm to identify six intensity clusters. The cluster labels are converted into an image in subject space as binary first pass segmentations. This is followed by the generation of a group average probability map or prior map for each cluster by warping the first pass binary segmentations into a common space using SPM's DARTEL “create template algorithm” (Step 8) which represents the first step of the last module or “final segmentation”, that is, the generation of probabilistic group averages to be used as priors to refine the segmentation outputs. The transformation matrix from this step was inverted and used to warp the probabilistic group averages into the subject/MNI space. The information from the priors was combined with the distance information from the clustering step which allowed to clean‐up voxels assigned to a cluster not consistent with the probability information and to convert the binary first pass segmentation into a probabilistic final segmentation (Step 9). Please see Methods for more details

Please see Figure 1 for an overview of the segmentation workflow.

### 2.4 Preprocessing

“Unified segmentation” as implemented in SPM12 (https://www.fil.ion.ucl.ac.uk/spm/software/) was used to remove a residual bias from the UNI and T1_map images and to obtain CSF, gray, and white matter tissue maps from the UNI image. The gray matter map was spatially normalized into the MNI space using SPM12's “normalize” function and template, and the forward and inverse transformations of this step calculated. The former was applied to all outputs (bias corrected UNI, T1_map, tissue maps, and brain tissue mask derived from combining the gray and white matter tissue maps).

The next step was to enhance the gray/white matter contrast in the bias‐corrected UNI and T1_map by rescaling them using a modified version of the linear scaling procedure described by Ganzetti, Wenderoth, and Mantini (2014). In contrast to Ganzetti et al., Ref1 and Ref2 were not obtained from nonbrain tissue regions of the ICBM152 template but fixed at Ref1 = 100 and Ref2 = 20 for both field strengths. The values for Ref1 and Ref2 had been experimentally determined by exploring a range of values in three 3 T and three 7 T images and maximizing the enhancement of gray/white matter contrast while minimizing the number of voxels with negative intensities (see later). The CSF map and the white matter tissue map were thresholded at 0.9 to identify voxels with high probability to be either CSF voxels or white matter voxels. The intensities of these high probability CSF and white matter voxels were extracted from each subject's UNI and T1_map and the modes of their intensity histograms determined after excluding voxels corresponding to vessels whose intensity was either below the 1st percentile (T1_map) or above the 95th percentile (UNI). The histogram modes were used to rescale the UNI and T1_map using the following formulas.

#### 2.4.1 T1‐weighted image

$$\begin{array}{l} \text{UNIfact}=\mathrm{abs}\left(\left(\mathrm{Ref}1-\mathrm{Ref}2\right)/\left(\mathrm{wm}\_\mathrm{UNI}\_\text{Mode}-\mathrm{csf}\_\mathrm{UNI}\_\text{Mode}\right)\right);\\ \text{UNIshift}=\mathrm{abs}(\left(\left(\left(\mathrm{wm}\_\mathrm{UNI}\_\text{Mode}*\mathrm{Ref}2\right)-\left(\mathrm{csf}\_\mathrm{UNI}\_\text{Mode}*\mathrm{Ref}1\right)\right)*2\right)/\left(\;\mathrm{wm}\;\_\;\mathrm{UNI}\;\_\;\text{Mode}\;-\;\mathrm{csf}\;\_\;\mathrm{UNI}\;\_\;\text{Mode}\;\right)\;)\;;\\ \;\mathrm{UNI}\;\_\mathrm{cal}=\left(\mathrm{UNI}*\text{UNIfact}\right)-\text{UNIshift}; \end{array}$$

wm_UNI_Mode, mode of histogram from voxels with more than 90% probability to be white matter, csf_UNI_Mode, mode of histogram from voxels with more than 90% probability to be CSF voxels, UNIfact, scale factor for UNI by which the original intensity range is reduced, UNIshift, distance by which the intensity histogram is moved towards the left, abs, absolute. UNI_cal, rescaled MP2RGAE_UNI image.

#### 2.4.2 T1_map

$$\begin{array}{l} T1\text{fact}=\mathrm{abs}\left(\left(\mathrm{Ref}2-\mathrm{Ref}1\right)/\left(\mathrm{wm}\_T1\mathrm{map}\_\text{Mode}-\mathrm{csf}\_T1\mathrm{map}\_\text{Mode}\right)\right);\\ T1\text{shift}=\mathrm{abs}(\left(\left(\mathrm{csf}\_T1\mathrm{map}\_\text{Mode}*\mathrm{Ref}2\right)-\left(\mathrm{wm}\_T1\mathrm{map}\_\text{Mode}*\mathrm{Ref}1\right)\right)/\left(\mathrm{wm}\_T1\mathrm{map}\_\text{Mode}-\mathrm{csf}\_T1\mathrm{map}\_\text{Mode}\right));\\ T1\_\mathrm{map}\_\mathrm{cal}=\left(T1\_\mathrm{map}*T1\text{fact}\right)–T1\text{shift}; \end{array}$$

wm_T1map_Mode, mode of histogram from voxels with more than 90% probability to be white matter, csf_T1map_Mode, mode of histogram from voxels with more than 90% probability to be CSF voxels, T1fact, scale factor for T1_map by which the original intensity range is reduced, T1shift, distance by which the intensity histogram is moved towards the left, abs, absolute, T1_map_cal, rescaled T1_map image.

**Figure 2:**
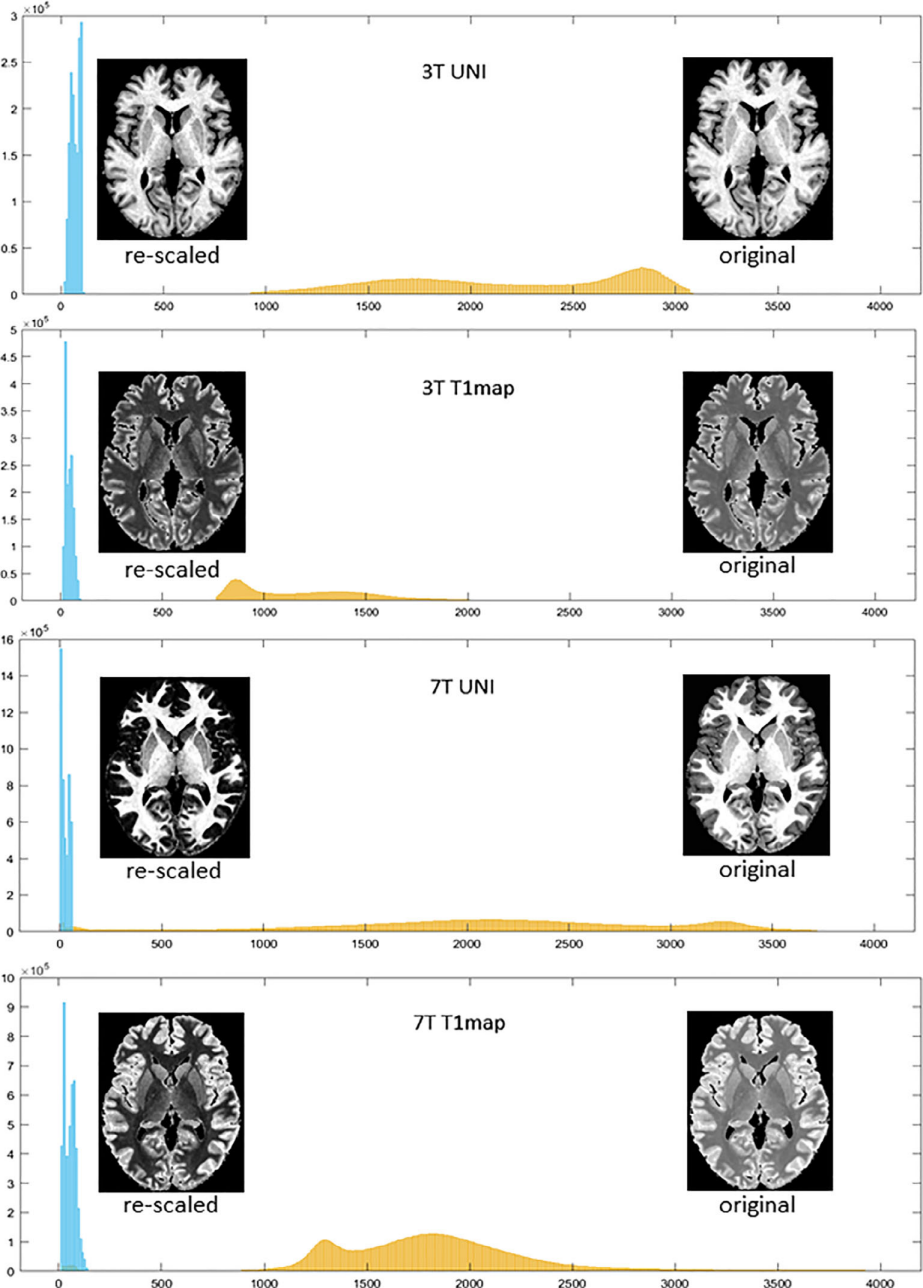
Image rescaling. Rescaling of UNI and T1_map. First and second row, 3 T image, third and fourth row 7 T images. Yellow intensity histogram before rescaling, blue histogram after rescaling

The rescaling introduced brain tissue voxels with negative intensities in the UNI and T1_map. These negative voxels were identified and replaced with the mean of the intensities of non‐negative first‐order neighborhood voxels. Figure 2 illustrates the effect of the rescaling on the histogram and the intensities of the UNI and T1_map. Table 1 summarizes the impact of the rescaling on gray/white matter contrast at 3 and 7 T in different brain regions.

**Table 1:** Mean (*SD*) gray/white contrast in original and rescaled images

Field strength	Image	Con hippo/temp WM	Con PAG/cerebel ped
3T	UNI orig	1.74 (0.15)	1.34 (0.09)
	UNI cal	3.64 (1.95)	2.01 (0.94)
	T1_map orig	1.63 (0.14)	1.35 (0.09)
	T1_map cal	2.78 (0.91)	2.07 (0.69)
	RATIO	11.28 (8.65)	4.39 (3.19)
7T	UNI orig	2.22 (0.31)	1.43 (0.16)
	UNI cal	6.06 (2.59)	2.00 (0.54)
	T1_map orig	1.64 (0.12)	1.33 (0.07)
	T1_map cal	2.72 (0.76)	1.94 (0.43)
	RATIO	16.26 (8.15)	3.87 (1.71)

Abbreviations: Con, contrast; hippo, hippocampus head, temp WM, white matter temporal stem PAG, periaqueductal gray; cerebel ped, cerebellar peduncle orig, original intensities; cal, rescaled intensities; UNI, T1 weighted image; T1_map T1 relaxation map; RATIO, ratio between UNI_cal and T1_map_cal.

The next step was to calculate the UNI_cal/T1_map_cal image or RATIO image from the rescaled UNI_cal and T1_map_cal. Voxels in the RATIO image whose intensity exceeded the 99th percentile were replaced with the mean of the intensities of the first‐order neighborhood voxels with intensities at or below the 99th percentile. Combining the rescaled UNI_cal and T1_map_cal in this way further increased the gray/white matter contrast (Figure 1, Steps 5 and 6, and Table 1). A brainstem/thalamus label in MNI space was generated from the 2009 ICBM 152 T1 atlas to extract the brainstem/thalamus images (bs) from the calibrated whole brain UNI_cal, T1_map_cal and RATIO images.

### 2.5 First‐pass binary brainstem segmentation and prior generation

A subject‐specific binary brainstem tissue mask was generated by thresholding the bs_UNI. This mask was used to extract the tissue intensities from each subject's bs_UNI, bs_T1_map and bs_RATIO image. The intensities from each image type were converted into z‐scores that were supplied to the k‐means clustering algorithm implemented in MATLAB 9.4 (The Math Works, Natick, MA; number of clusters *n* = 6, squared Euclidian distance function, maximum number of iterations = 1,000, replicates = 100). The optimal number of clusters *n* = 6 had been determined experimentally by exploring the range from 4 (no of tissue components identified by Lambert et al. (2014)) to 8 in three subjects. With *n* = 6 clusters, one cluster corresponded to the outer brainstem boundaries and the remainder highlighted different internal brainstem structures when displayed in image space (cf Figure 1, Step 7). The cluster centroid information of each subject was matched to the centroid information of a randomly selected reference subject and the cluster numbering accordingly changed to ensure a consistent cluster numbering/centroid assignment across different subjects.

Each subject's binary image space representations of these six clusters were simultaneously registered to six common group averages for each field strength (3 T average: 50 subjects 1 mm isotropic resolution; 7T average: 46 subjects, 0.7 mm isotropic resolution) using the “create template” function of DARTEL (Ashburner, 2007) as implemented in SPM 12 (Figure 1, Step 8). An intensity close to 1 in the group average of cluster X indicates a high probability that a voxel has been assigned to cluster X in the group average and an intensity close to 0 a low probability that it has been assigned to cluster X. The transformation matrices generated for each subject during this process were inverted and applied to the group average of each cluster to project the latter into each subject's cluster image space.

### 2.6 Final segmentation

Using the information from the probabilistic group averages in subject space and the centroid distances, each brainstem voxel in the individual subject was assessed for consistency, that is, cluster assignment based on centroid distance is identical to cluster assignment based on highest probability in probabilistic group averages. Voxels with inconsistent assignments were reassigned to the cluster suggested by the probabilistic group averages (alternate cluster) if they met one of the following conditions. (a) Probability that voxel belongs to the alternate cluster is ≥0.75. (b) Probability that voxel belongs to the alternate cluster is higher by ≥0.20 than probability to belong to original cluster. Finally, the binary cluster assignments for each subject were converted into probabilistic assignments by multiplying them with the corresponding probabilistic group average weighted by the standardized centroid distance information for this subject. The centroid distance information was standardized by scaling the original distances to values between 0 and 1 so that the voxel closest to the cluster centroid had the value closest to 1 and the voxel with the greatest distance a value closest to 0.

### 2.7 Rationale and evaluation of segmentation approach

The use of the z‐transformed intensities from all three bs images in the cluster analysis may at first seem redundant given their highly correlated intensity profiles. It was based on the following reasoning: The two gradient echo images acquired by the MP2RAGE sequence differ not only re inversion time but also how each voxel is affected by noise. The bs_UNI, bs_T1map, and bs_RATIO images represent essentially different ways to combine the two MP2RAGE gradient echo images and each of them has a slightly different impact on image noise. Furthermore, the rescaling not only enhances gray/white contrasts but also replaces intensity outliers likely to correspond to noise voxels with intensity values derived from neighboring non‐noise voxels. As a consequence, a cluster analysis with the bs_UNI as only input would for example assign some of these noise affected voxels to the gray matter structures captured by cluster X if they are intensity‐wise a match. Since this decision is solely based on intensities, these noise voxel might even get high values in a silhouette analysis despite clearly not belonging to the X gray matter structures based on their spatial relationship to these gray matter structures. However, because of the randomness of the noise, the different ways to combine the two images, and the rescaling, these noise voxels are less likely to behave in the same way, that is, to be miss‐classified as X gray matter structure voxels with high silhouette values, if just the bs_ratio or just the bs_T1_map image are used for the cluster analysis. Instead another set of noise voxels will be misclassified in these images. In contrast, noise‐free voxels belonging to the gray matter structures captured by cluster X are expected to behave in a consistent way, that is, to be assigned to cluster X with a relatively high silhouette value regardless which one of the three images was used in the cluster analysis. Using all three bs images together in the cluster analysis was therefore expected to take optimal advantage of the different behavior of non‐noise and noise voxels by assigning a high percentage of the former with high silhouette values to the cluster X and reduce the number of the latter by either assigning them to an alternative and ideally correct cluster or to assign them to cluster X with a low silhouette value that would translate in a low probability value in the final segmentation.

The correctness of the rationale outlined in the previous paragraph was tested by repeating the steps described in the section “first pass segmentation and prior generation” with each image type as sole input in the cluster analysis (number of clusters *n* = 6, squared Euclidian distance function, maximum number of iterations = 1,000, replicates = 100) and with the combinations bs_UNI/bs_T1_map and bs_RATIO/bs_T1_map as inputs. The following two tests were performed: (a) Silhouette plots were generated and the percentages of voxels with a silhouette value exceeding 0.6 for each cluster with exception of partial volume Cluster 2 calculated. Their mean or “low silhouette index” was used to assess the overall performance of the six variants (all three images, combination of two, each image separately) in each 3 T and each 7 T subject. If the assumption regarding the behavior of noise voxel is correct then the three image approach was expected to have the highest low silhouette indices reflecting the successful “downgrading” of noise voxels, the single image approaches the lowest low silhouette indices and the two image approaches intermediate low silhouette indices. (b) The percentage of voxels that was incorrectly assigned to a cluster based on their location in the image in relation to the structures identified by this cluster was calculated by projecting the priors back into the subject space and thresholding them at 0.3, counting all voxels in the first pass segmentations not included in the resulting mask and comparing that number to all voxels assigned to this cluster. The mean percentage over all clusters or “miss‐classification index” was calculated to obtain a measure of “misclassification” for each approach. If the assumptions regarding the behavior of noise voxels made in the previous paragraph is correct, the three image approach should show the lowest miss‐classification indices of all approaches.

### 2.8 Regional analysis

Two types of region of interest (ROI) analyses were performed: (a) Cluster ROI analysis: Each subject's first pass segmentation outputs were used to extract the mean intensities from their bs_ UNI, bs_T1_map, and bs_RATIO images with the goal to characterize the intensity properties of the clusters and by extension the intensity properties of the tissue types identified by them. (b) Anatomical ROI analysis: The probabilistic group averages of the six clusters were thresholded at 0.3 and used to manually delineate 27 regions of interest using the brainstem atlas from Naidich and Duvernoy (Naidich et al., 2009) as reference. The following regions of interest (rois) were labeled: Left and right corticospinal, frontopontine and parietotemporopontine tracts, bilateral medial lemniscus, inferior, middle and superior cerebellar peduncle, ncl. raphe dorsalis, raphe magnus, ncl reticularis medullae oblongata, parvocellularis and gigantocellularis, pedunculopontinus and cuneiformis, ncl. pontis oralis, ncl. pontis tegmenti caudalis, locus coeruleus, ncl. parabrachialis, ncl. tractus solitarius, ncl. olivaris inferior, pontine nuclei, ncl. ruber, substantia nigra, periaqueductal gray, ventral tegmental area (see Figures 1 and 6). The manual labeling was done using the 3 T priors. The rois were then resampled to the 7 T space and resolution, their accuracy checked and manually edited if necessary. For the analysis each set of rois was projected back into subject space by applying the inverse of the transformation matrices generated during preprocessing. These rois were used to extract the mean intensities values from these regions from the probabilistic group averages and each subject's bs_UNI, bs_T1_map, and bs_RATIO image with the goal to investigate the intensity properties of these rois and how they are influenced by age.

### 2.9 Deformation‐based morphometry

Each subject's six probabilistic segmentation maps in MNI space were warped into a common space resulting in a brainstem template using the “create template” function of DARTEL. The resulting transformation matrices were converted into Jacobian maps. The unsmoothed Jacobian maps were used to test for age‐related brainstem loss by comparing the two age groups. The same was done with the unsmoothed bs_UNI, bs_T1_map, and bs_RATIO images of each subject. Using the probabilistic maps and brainstem grayscale images in the MNI space instead of subject space eliminates differences due to head size and gross shape differences and allows for a better detection of intensity/shape differences of smaller structures.

### 2.10 Statistical analysis

T‐tests (for normal distributed data) Wilcoxon signed rank tests (no normal distribution) at *p* < .05 were used to test for segmentation performance testing, group differences, for example, 3 T versus 7 T measurements or young vs. old ROI intensities. False Discovery Rate (FDR) q = .05 was used to correct for multiple comparisons for the analyses using ROI‐based intensities and Family‐wise‐error rate *p* < .05 was used to correct for multiple comparisons for voxel‐based analyses.

## 3 RESULTS

### 3.1 Segmentation performance

Table 2 lists the mean and standard deviations of the low silhouette indices and the miss‐classification indices for each tested image combination and each field strength. Figure S1 shows the priors generated by each image combination. The findings are in agreement with the rationale for the three image cluster analysis outlined in the methods section, that is, the three image approach has consistently high low silhouette indices indicating a “downgrading” of miss‐classified noise voxels, the lowest miss‐classification indices indicating an “elimination” of miss‐classified noise voxels that have also significantly lower standardized distances. Furthermore, the cluster analysis using all three image types also clearly outperformed the first pass segmentations based on a single image or combinations of two images on visual inspection.

**Table 2:** Segmentation performance summary

Field strength	Measure	All three images	UNI	RATIO	T1_map	UNI/T1_map	RATIO/T1_map
3 T	Low silhouette index	31.6 (2.7)	28.1 (0.4)*	27.1 (0.4)*	28.7 (1.8)*	30.8 (2.2)*	30.2(2.2)*
	Miss‐classification index	29.2 (3.4)	36.9 (6.4)*	33.9 (4.5)*	43.5 (7.1)*	38.9 (7.8)*	31.5 (5.6)*
	Miss‐classification stand distance	0.99465 (0.00046)	0.99675 (0.00026)*	0.99529 (0.00037)*	0.99570 (0.000679)*	0.99513 (0.00061)*	0.99524 (0.00040)*
7 T	Low silhouette index	30.4 (1.7)	28.3 (0.4)*	26.S (0.5)*	28.6 (1.8)*	31.3 (1.8)*	29.1 (1.9)*
	Miss‐classification index	29.3 (3.1)	37.0 (4.5)*	32.6 (4.4)*	33.2 (4.8)*	33.4(4.9)*	31.0(3.2)*
	Miss‐classification stand distance	0.995427 (0.000475)	0.997235 (0.000383)*	0.995468 (0.000228)*	0.996461 (0.000522)*	0.995992 (0.000476)*	0.996042 (0.000469)*

*Notes*: Low silhouette index percentage of voxels with silhouette values below 0.6; % miss‐classification index percentage voxels outside structure detected by cluster, miss‐classification stand distance, mean standardized (refer text for definition) distance of miss‐classified voxels.*p < .05 compared to all three images. Indices are given as means and standard deviations in brackets.

### 3.2 Tissue types in probabilistic group averages

**Figure 3:**
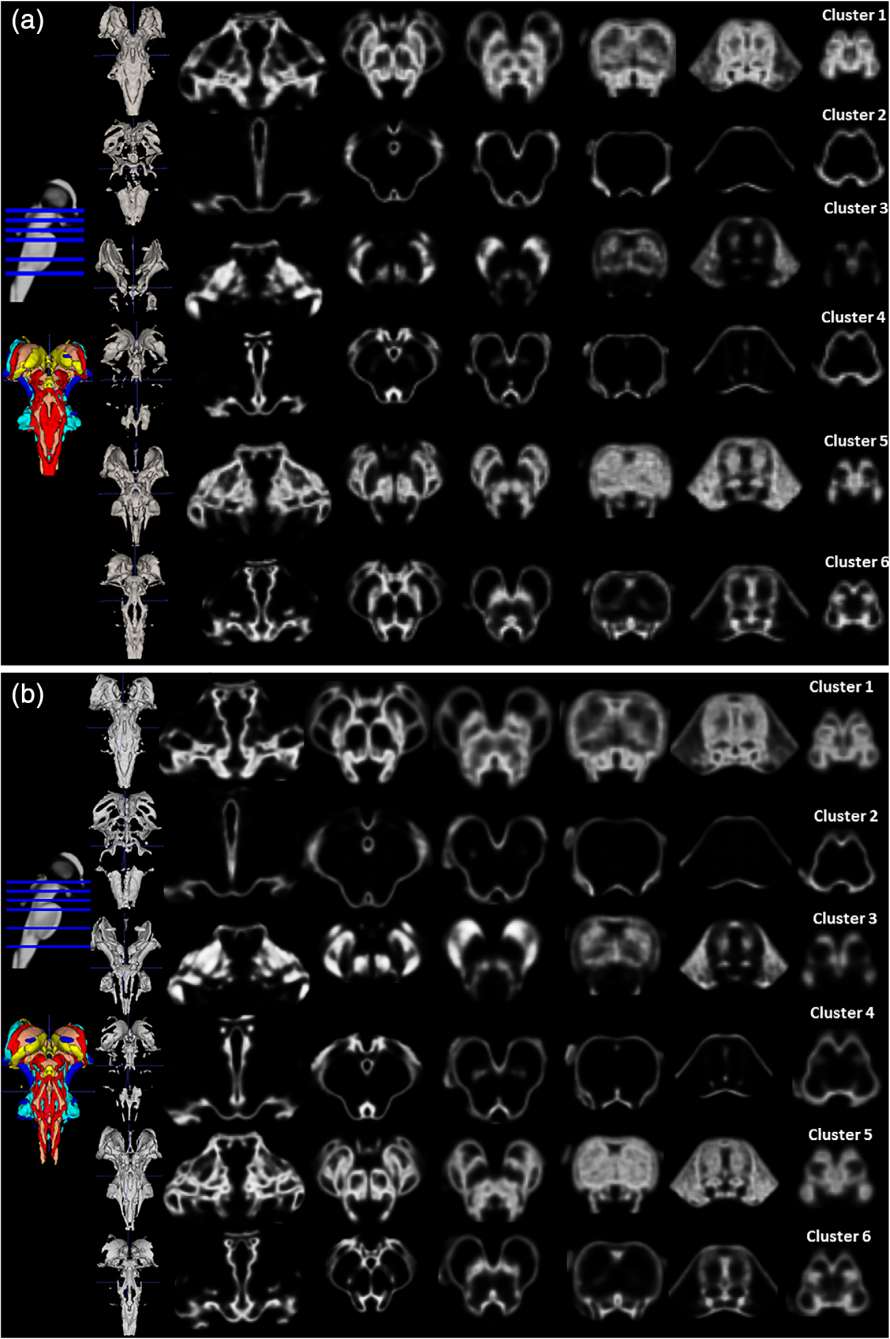
Probabilistic group averages at 3 T (a) and at 7 T (b). Left side of the panel, sagittal scout image indicating the localization of the cluster cross‐sections displayed on the right side, a 3D composite reconstruction and 3D reconstructions of individual cluster/tissue types

**Figure 4:**
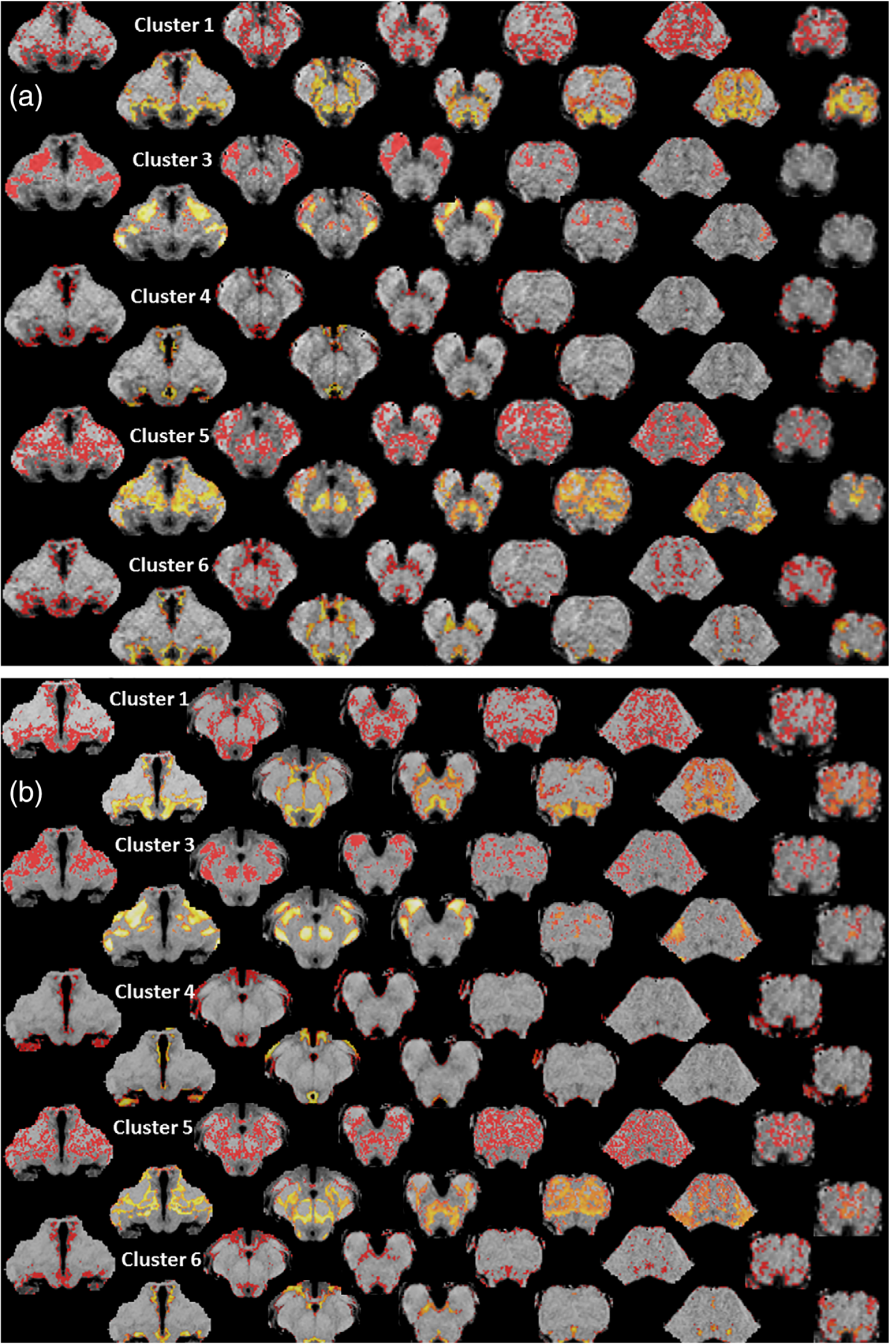
First pass and final segmentations at 3 T (a) and at 7 T (b). The localization of the cross‐sections corresponds to that shown in Figure 3. The segmentations are overlaid on the subjects bs_UNI, the first pass segmentation or binary image space representation of each cluster is in red, the final probabilistic segmentation is in “hot” with the intensity representing the voxel's probability to belong to the cluster

Figure 3a,b depicts the six unthresholded probabilistic group averages for the 3 T data and the 7 T data. Figure 4a,b provides an example of the segmentation quality in individual subjects. Using the brainstem atlas from Naidich and Duvernoy as reference, Cluster 1 corresponds to brainstem gray matter structures at both field strengths. It included the substantia nigra (compacta), the superior and inferior colliculi, the pontine nuclei and the reticular nuclei of the mesencephalon, pons, and medulla. Cluster 2 is a “partial volume” cluster that consisted of voxels at the CSF/tissue boundary of the brainstem. Cluster 3 voxels belong mostly to white matter tracts, in particular to the frontopontine and corticonuclear tract at the mesencephalon level, to the corticonuclear and corticospinal tract, the medial lemniscus and cerebellar tract at the pons level and to the corticospinal tract and the medial lemniscus at the medulla level. Cluster 3 also delineates the ncl. ruber. Cluster 4 corresponds to the periaqueductal gray and raphe. Cluster 5 identified white matter tract voxels and voxels in gray/white matter transition zones. At the level of the mesencephalon, the label included voxels within the corticospinal tract, the superior cerebellar peduncle and its decussation, and the ncl. ruber. At the level of the pons, Cluster 5 labeled voxels within the corticospinal and corticonuclear tract, the cerebellar peduncle and the medial lemniscus. At the medulla level, it high‐lightened the tectospinal and trigeminothalamic tract and the longitudinal fascicle. Cluster 6 again identified gray matter voxels. At the level of the mesencephalon, it highlighted the ncl. Dorsalis raphe, the ventral tegmental area and the substantia nigra (reticulata) and at the level of the pons, the locus coeruleus, ncl. abducens and raphe magnus. At the level of the medulla Cluster 6 outlined the ncl. olivaris inferior and the ncl. tractus solitarius.

### 3.3 Comparison of probabilistic group averages at 3 T and 7 T

**Figure 5:**
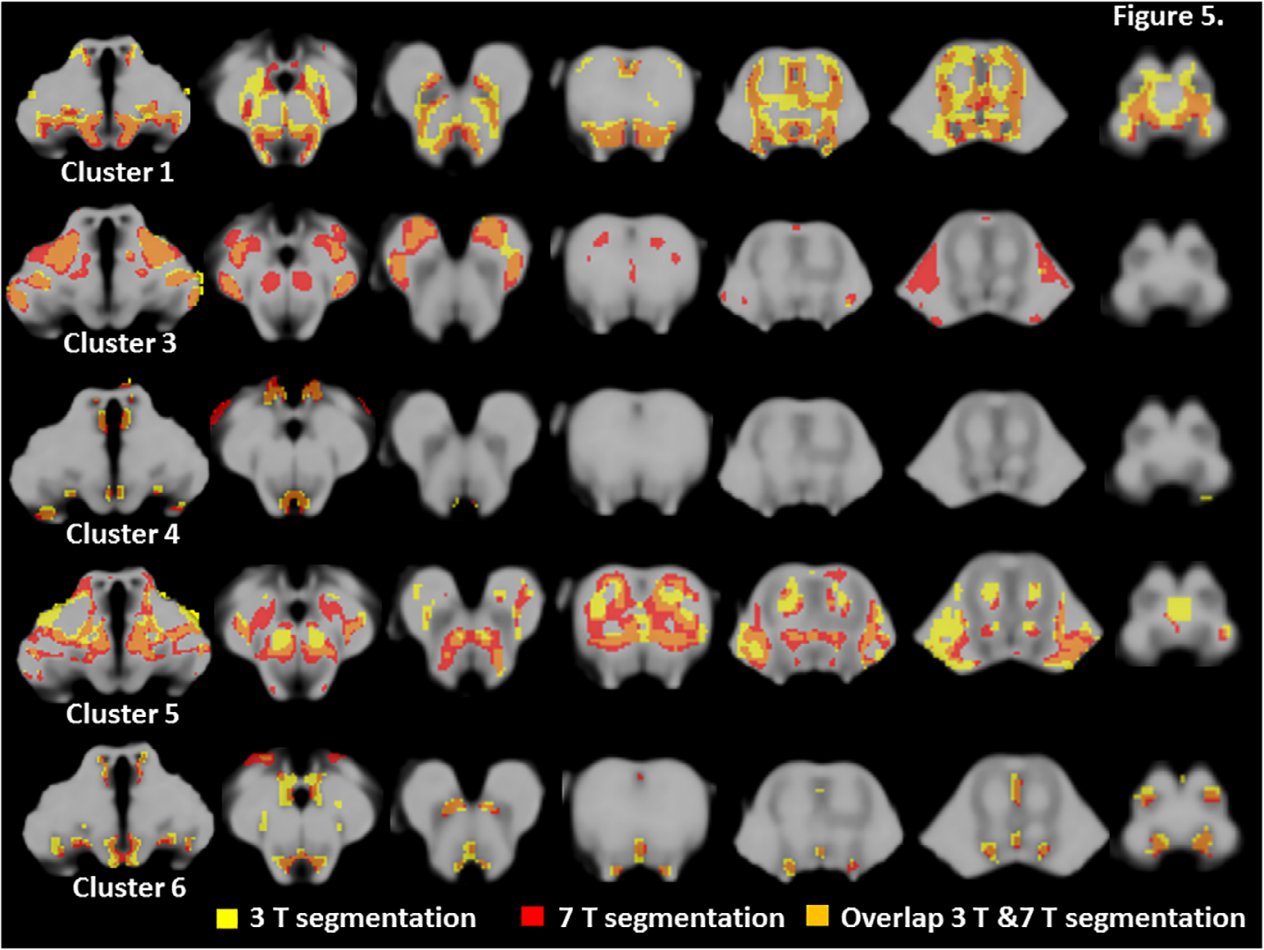
Direct comparison/overlay. Overlay of the thresholded (≥0.3) probabilistic 3 T (yellow) and 7 T (red) group averages. Regions that are assigned to the same cluster by both field strengths appear in orange

Subtle differences between the 3 T and 7 T probabilistic averages become apparent in the qualitative side‐by‐side comparison. Figure 5 suggests that the gray matter segmentation in Clusters 1 and 6 is more prominent and also coarser at 3 T than at 7 T and that the white matter segmentation (Cluster 5, but also Cluster 3) is more prominent and detailed at 7 T than at 3 T. The difference is particularly obvious in the substantia nigra and ncl. ruber. At 3 T, the substantia nigra is distributed between Clusters 1 and 6. This is also the case at 7 T but in addition a small region that overlaps with the localization of a myelinated cluster within the substantia nigra seen in histological preparations (Massey et al., 2017) is assigned to white matter Cluster 5. The ncl. ruber is ill defined and “split” between Clusters 3 and 5 at 3 T but sharply defined and fully depicted in Cluster 3 at 7 T. The assignment of the ncl. ruber—an iron rich gray matter structure—to the white matter Clusters 3 and 5 is unexpected but could be explained by the presence of myelinated fibers from the superior cerebellar peduncle, oculomotor nerve, and habenulointerpeduncular tract that are passing through the ncl. ruber. The clear separation between white matter Clusters 3 and 5 at 7 T compared to 3 T is not restricted to the ncl. ruber though but affects other white matter structures as well, for example, corticospinal and corticonuclear tract in Cluster 3 at 7 T at the pons level that is missing at 3 T in Figure 5.

For the quantitative comparison, regional information from each probabilistic group average was extracted using the anatomical ROIs. The mean probabilities were generally slightly higher at 3 T than at 7 T [mean (*SD*) over all 27 regions: 0.51 (0.08) vs.0.47 (0.07), *p* = .03]. Grouping ROIs according to tissue type, that is, gray matter ROIs (*n* = 17) and white matter ROIs (*n* = 10) showed higher gray matter probabilities in gray matter ROIs at 3 T compared to 7 T [0.55 (0.09) vs. 0.46 (0.07), *p* = .004] and slightly lower white matter probabilities in white matter ROIs at 3 T compared to 7 T [0.46 (0.02) vs. 0.48 (0.07) *p* = .46]. Grouping anatomical ROIs by brainstem level, that is, mesencephalic ROIs (*n* = 10), medulla ROIs (*n* = 6) and ROIs spanning more than one level, for example, tracts or pons ROIs (*n* = 11) and comparing probabilities by level showed no difference between 3 and 7 T for mesencephalic ROIs [0.52 (0.9) vs. 0.49 (0.7) *p* = .28] and tract/pons ROIs [0.48 (0.06) vs. 0.47 (0.8), *p* = .66] but lower probabilities at 7 T for ROIs in the medulla [0.56 (0.08) vs. 0.44 (0.06), *p* = .02], indicating the existence of a rostral–caudal gradient of tissue probabilities at 7 T but not at 3 T.

### 3.4 Comparison of regional intensities and age effects

**Figure 6:**
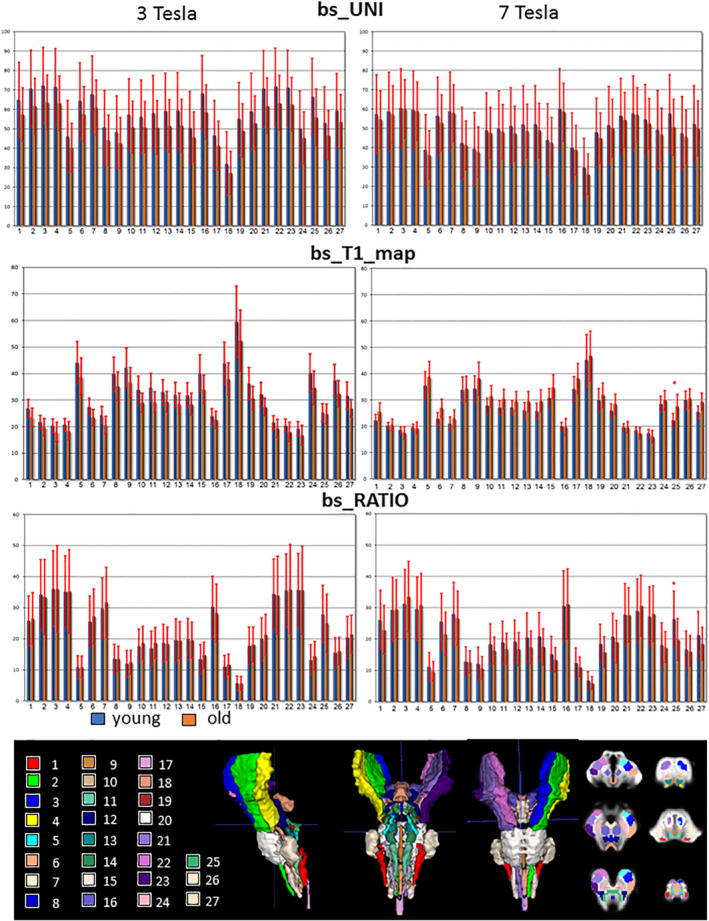
Regional intensities in anatomical regions of interest (ROI). Mean image intensities in 27 brainstem ROIs, left panel 3 T, right panel 7 T, for the two age groups, young (20–45 years of age), blue; old (45–80 years of age, orange. Regions of interest: 1. inferior cerebellar peduncle; 2. left corticospinal tract; 3. left frontopontine tract; 4. Left parietotemporopontine tract; 5. locus coeruleus; 6. medial lemniscus; 7. middle cerebellar peduncle; 8. ncl. raphe dorsalis; 9. ncl. raphe magnus; 10. ncl. reticularis medullae oblongatae centralis; 11. ncl. reticularis parvocellularis and gigantocellularis; 12. ncl. reticularis pedunculopontinus and cuneiformis; 13. ncl. reticularis pontis oralis; 13. ncl. reticularis pontis oralis; 14. ncl. reticularis pontis tegmenti caudalis; 15. ncl. olivaris inferior; 16. ncl. ruber; 17. ncl. tractus solitarius; 18. periaqueductal gray; 19. ncl. parabrachialis; 20. pontine nuclei; 21. right corticospinal tract; 22. right frontopontine tract; 23. right parietotemporopontine tract; 24. substantia nigra; 25. superior cerebellar peduncle; 26. ventral tegmental area; 27. ventrolateral medulla

Figure 6 depicts the average intensities of 27 anatomical ROIs in the rescaled images in young and old subjects at 3 and 7 T. The intensity distribution across different brainstem regions at both field strengths is very similar but in contrast to the behavior of the original images the intensities are slightly lower in the rescaled 7 T images compared to rescaled 3 T images. This reflects the larger intensity range of both original images at 7 T that resulted in lower scaling and shift factors compared to 3 T: Mean (*SD*) UNIfact and UNIshift were 0.04 (0.009) and 40.5 (27.1) at 3 T and 0.03 (0.002) and 29.8 (10.2) at 7 T. Mean (*SD*) T1fact and T1shift were 0.08 (0.029) and 45.2 (25.2) at 3 T and 0.05 (0.02) and 37.8 (21.0) at 7 T.

Except for a lower intensity of the superior cerebellar peduncle in the bs_T1_map that was associated with a higher intensity in the bs_RATIO at 7 T, none of the age related intensity differences was significant after FDR correction. Interestingly, the young group had consistently higher bs_T1_map intensities than the old group at 3 T but lower bs_T1_map intensities than the old group at 7 T. The same phenomenon was also observed in the non‐rescaled T1 maps (data not shown) which indicates that it is not caused by the rescaling.

### 3.5 Comparison of deformation‐based morphometry findings

**Figure 7:**
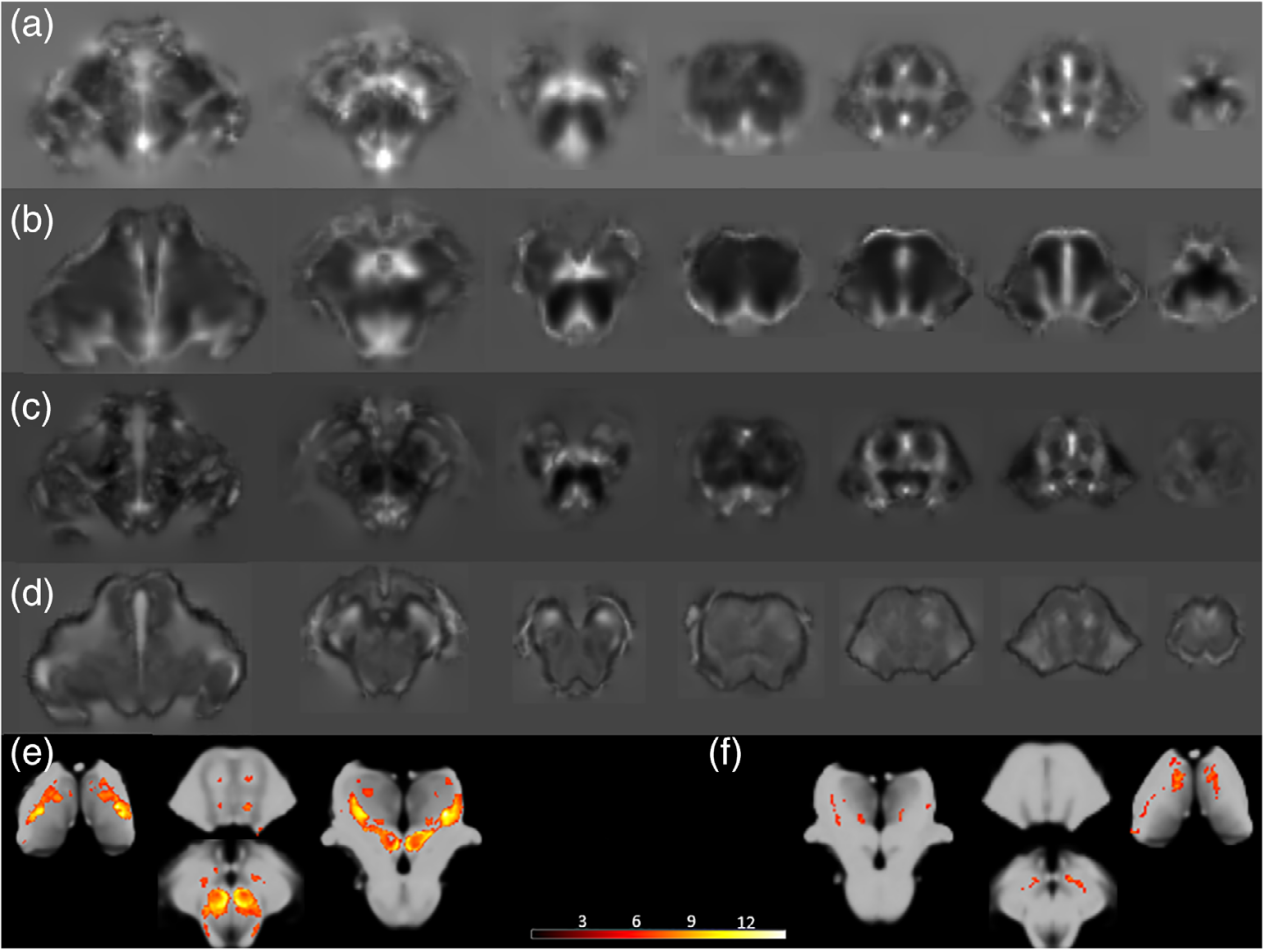
Jacobian determinants and age‐associated volume loss at 3 T and at 7 T. (a) Jacobian determinants derived from the transformation matrix generated by warping the six 3 T final tissue segmentation outputs onto the brainstem tissue template. (b) Jacobian determinants derived from the transformation matrix generated by warping the three rescaled brainstem images onto a grayscale template generated from all 50 3 T subjects’ grayscale brainstem images. (c) Jacobian determinants derived from transformation matrix generated by warping the six 7 T final tissue segmentation outputs onto the brainstem tissue template. (d) Jacobian determinants derived from transformation matrix generated by warping the three rescaled brainstem images onto a grayscale template generated from all 46 7 T subjects’ grayscale brainstem images. (e) Age related volume loss (young (20–45 years) > old (45–80 years)) based on 7 T tissue segmentations. (f) Age related volume loss (young [20–45 years] > old [45–80 years]) based on 3 T tissue segmentations

Figure 7 depicts the Jacobian determinants derived from warping an individual subject's six probabilistic segmentation outputs onto the brainstem template generated by the DARTEL “generate template” algorithm and the Jacobian determinants generated by warping the same subject's three grayscale images (bs_UNI, bs_T1_map, and bs_RATIO) onto the grayscale brainstem template generated by DARTEL. The Jacobian determinants derived from warping the segmentation outputs show more details of the internal structure than the Jacobian determinants derived from the grayscale images.

Deformation‐based morphometry using the Jacobian determinants derived from the final, probabilistic segmentations at 7 T showed age‐associated volume loss in the region of the ventral lateral thalamic nucleus that extended into the superior cerebellar peduncle including its decussation and into the ascending uncinate tract at the level of the mesencephalon. At the level of the pons, age‐associated volume loss was seen in the region of the corticospinal tract and the medial lemniscus. There were no significant age‐associated volume losses when this analysis was done with Jacobian determinants derived from the three rescaled 7 T grayscale images.

Using the Jacobian determinants derived from the six probabilistic 3 T segmentations, highlighted small regions of age‐associated volume loss in the anterior and ventral lateral thalamic nucleus and in the substantia nigra. Again, there were no significant age‐associated volume losses when this analysis was done with the Jacobian determinants derived from the three rescaled 3 T grayscale images.

## 4 DISCUSSION

This study had several major findings. (a) The brainstem segmentation method described in this article exploits the inherent strengths of the MP2RAGE sequence, that is, the simultaneous acquisition of a T1 weighted image with excellent gray/white contrast and of a T1 relaxation map suitable for myelin mapping. A linear intensity rescaling reduces the intensity ranges and magnitudes of the two MP2RAGE images and thereby nearly doubles their gray/white contrasts independently of the field strength. These rescaled images are used to calculate a ratio image whose gray/white contrast at the cortical and brainstem level is again greater than that of the two input images. A k‐means cluster analysis identifies six intensity/tissue clusters using the z‐transformed brainstem voxel intensities from these three images as inputs. Combining the centroid distance information from the clustering step with the information from the six probabilistic group averages or priors converts the binary segmentation into the final probabilistic segmentation. (b) The segmentation method works with 7 T but also with 3 T MP2RAGE images, that is, is not dependent on the availability of a high field magnet. (c) Although single subject segmentations and probabilistic group averages/priors were similar at 3 and 7 T on visual inspection, there were subtle differences in the quantitative comparison that affected the distribution of age‐related brainstem volume losses. Additional investigations will be necessary to fully understand the nature of these differences and to refine the acquisition/processing parameters to optimize the information obtained from the MP2RAGE.

Taken together, this article presents a new brainstem segmentation approach that is based on a sequence that can be easily implemented on a clinical 3 T magnet and therefore has the potential to be used for routine screening of brainstem pathologies.

On visual inspection (Figure 3a,b) the 3 and 7 T probabilistic group averages appear to be very similar. Each cluster highlights the same distinct brainstem structures that belonged predominantly to either gray (Clusters 1, 4, and 6) or white matter (Clusters 3 and 5) or represented the boundary between brain tissue and csf (Cluster 2). The probabilistic group averages have also a striking resemblance to the probability maps shown by Lambert et al. (2014). Lambert and co‐workers acquired a 3D multi‐echo FLASH sequence at 3 T with a total acquisition time of 1 hr 15 min in 34 subjects to generate magnetization transfer, proton‐density weighted, R1 and R2* maps with a 0.8 mm isotropic resolution and used a modified multivariate mixture of Gaussians to perform a multichannel brainstem segmentation. Tissue class 1 in Lambert et al. (2014) corresponds to Cluster 6 in this study, Tissue class 2 to Cluster 1, Tissue class 3 to Cluster 4, and Tissue class 4 to a combination of Clusters 5 and 3. Lambert and co‐workers also identified a tissue class made up from voxels at the tissue/csf boundary that corresponds to Cluster 2 in this study. The similarity between the probabilistic averages of these two studies and to brainstem structures identified in ex vivo ultra‐high field studies (Naidich et al., 2009) raises the confidence that the approach proposed here is indeed able to identify some of the more prominent features of the internal brainstem anatomy at the group and single subject level (Figures 3 and 4).

The quantitative comparison using the mean probabilities from each cluster extracted by the 27 anatomical ROIs found subtle differences between the 3 and 7 T brainstem segmentations. The mean probabilities in gray matter ROIs tended to be generally lower at 7 T than at 3 T and this difference was more pronounced in medulla ROIs than in mesencephalon ROIs. While there exist several possible explanations for the generally lower gray matter probabilities at 7 T, for example, number of scans for probability map generation 46 for 7 T but 50 for 3 T, higher resolution at 7 T reducing partial volume effects at the ROI edges, field strength specific behavior favoring gray matter intensities at 3 T and white matter intensities at 7 T, rescaling parameters not optimized for each field strength, and so forth, the more pronounced mesencephalon/medulla probability gradient at 7 T is very likely caused by a field strength inherent problem. The quality of the images derived from the MP2RAGE sequence is highly dependent on the efficiency of the initial adiabatic inversion pulse that must fulfill the adiabatic condition throughout the whole brain. Because of the increased B1 field inhomogeneities, this is harder to accomplish at 7 T particularly in brain regions covered by the lower end of the coil, that is, cerebellum and brainstem. Insufficient image quality in these regions caused by this issue was the reason that seven studies from the ATAG data set had to be excluded from processing. Although the remainder was deemed to be of sufficient quality, the signal/noise was slightly reduced in the medulla compared to the mesencephalon in some of the studies which affected the segmentation accuracy in this region. Optimizing the MP2RAGE sequence for brainstem imaging, for example, by improving the adiabatic pulse (Marques et al., 2010; Marques & Gruetter, 2013) or by the strategic placement of dielectric pads (O'Brien et al., 2014) should reduce the number of images lost due to insufficient data quality.

The differences described in the previous paragraph also affected the Jacobian determinant maps generated from the final segmentation outputs and by extension the localization of age‐related brainstem tissue loss. The maps generated from the 7 T segmentation were typically sharper and more detailed than those generated from the 3 T segmentations. It is not clear though to what degree this is due to the higher image resolution at 7 T or represents an inherent characteristic of the higher field strength. Comparing 7 T Jacobian maps of old subjects with those of young subjects identified volume losses in the thalamus, superior cerebellar peduncle and corticospinal tract. At 3 T, age‐related brainstem volume losses were generally less prominent than at 7 T and located in the thalamus and in the substantia nigra. Although it is important to keep in mind that some of the differences might just reflect the different study populations, it has to be pointed out that the greatest differences were found in the mesencephalon, that is, in the region with some of the most prominent differences in the 3 and 7 T probabilistic group averages. Furthermore, at 7 T age‐associated volume loss was mostly found in white matter structures but affected gray matter structures close to these white matter structures at 3 T which is in line with the differences of the gray/white segmentation at 3 T compared to 7 T that was discussed in previous paragraphs.

The study has several limitations: (a) The imaging data came from two data repositories that used standard implementations of the MP2RAGE sequence for the two field strengths. None of them had been optimized to visualize the brainstem. On the one hand, this supports the conclusion that it is possible to obtain good quality brainstem segmentations from images that could easily be implemented in routine clinical protocols. On the other hand, the different resolution and the different study populations prevented a more in‐depth investigation into possible causes for some of the differences seen at the two field strengths, for example, more prominent gray matter segmentation at 3 T and different behavior of the T1 relaxation map intensities in the old vs. young comparisons. (b) The same rescaling and segmentation routines were used for both field strengths, for example, parameters such as Ref 1 and Ref 2 were selected based on their ability to provide a good segmentation at both field strengths and not optimized for each field strength separately. It cannot be excluded that an optimization of the parameters for each of the field strengths could have reduced the differences between them. (c) The age distribution in the LEMON and ATAG data sets with peaks at 20–30 years and 50 years and older but only few or no middle‐aged participants did not allow for more sophisticated modeling of potential age effects. It cannot be excluded that this contributed to the different age effects at the two field strengths.

Taken together, although more work still needs to be done, the findings presented here suggest that it is possible to obtain a meaningful segmentation of internal brainstem structures using the MP2RAGE sequence. The MP2RAGE sequence is part of the 3 and 7 T Siemens neuro sequence package and has been implemented on Philips 7 T magnets for research purposes. The improved gray/white contrast due the inbuilt bias correction and the additional T1 map justify the slightly longer acquisition time (8–12 min vs. 6 min) of the MP2RAGE compared to a MPRAGE or other whole brain T1 weighted sequences that are routinely acquired in clinical imaging protocols. Together with a brainstem segmentation routine such as the one proposed here implemented for example on a cloud‐based computing platform and combined with a library of age‐adjusted normal ranges this sequence could provide a means to routinely investigate brainstem structures and thus eventually to diagnose diseases and conditions affecting the brainstem, for example, Alzheimer and Parkinson's disease, increased SUDEP risk in epilepsy, earlier and more accurately.

## CONFLICT OF INTEREST

The author has no conflicts of interest to declare in relationship to this work.

## Supporting information


**Figure S1** Panels (a) and (b) show the probabilistic segmentation maps or priors generated when using all three images as done in the manuscript or when only using one image type or a combination of two image types as input for the cluster analysis at 3 and 7 T. All prior images were thresholded to show only probabilities ≥0.3. The following qualitative criteria were used to assess the quality of the resulting priors: (1) Presence of clear probability gradients, that is, high probability (>0.9) in the center of the key structures recognized by each cluster and low probabilities (<0.3) at their boundaries. (2) Different structures are separated by the clusters, that is, are represented in one cluster and not distributed between two or more clusters that each show a faint imprint of the structure. (3) The quality of the segmentation of gray matter structures (Clusters 1, 4, and 6) is considered to be more important than that of white matter structures (Clusters 2 and 5). Using these criteria, the following observations can be made.
The three image‐based segmentations show good quality at both field strengths.The segmentations based on the UNI image as sole inputs do also perform well at both field strengths but the gray matter clusters are slightly less pronounced than that of the 3 image segmentations, that is, perform worse on criterion 1 than the 3 image approach.The same observations apply to the segmentations obtained with the ratio image as sole input at 3 T and 7 T. The exception is cluster 4 that shows more partial volume voxels (= miss‐classified voxels) in tissue/csf boundary regions then the 3 image versions. In addition to this, the white matter segmentations derived from the ratio image as sole input are less pronounced than in the 3 image version, that is, also perform worse on criterion 1 than the 3 image approach.The results obtained from the T1 maps as sole input differ between the field strengths. At 7 T, gray matter Clusters 1 and 2 are slightly less pronounced than those derived from the 3 image segmentation and gray matter Cluster 4 is only a faint imprint. In contrast, white matter Clusters 2 and 5 are very prominent. Cluster 5 includes voxels belonging to the pontine nuclei that are distributed between clusters 1 and 5, i.e., the segmentation performs worse on criterion 3. At 3 T, the exchange of voxels between gray matter cluster 1 and white matter cluster 5 goes in the opposite direction, that is, a large proportion of the voxels classified as white matter with the 3 image approach are identified as gray matter and assigned to cluster 1. The T1 map based Cluster 5 segmentation at 3 T is an obvious outlier and was not included into the calculation of the miss‐classification index etc reported in Table 2.Using T1 maps together with ratio images as inputs generates Clusters 1, 5, and 6 priors of good quality at both field strengths. Clusters 2 and 4 are slightly less pronounced than the corresponding three image priors.The segmentations based on T1 map and UNI images as input result in good quality segmentations for Cluster 1, and slightly more prominent segmentations for Clusters 3, 5 compared to the corresponding three image segmentations. Clusters 4 and 6 segmentations are slightly less prominent compared to the three image outputs at 3 T but well segmented at 7 T.
In summary, the three and two image approaches and particularly the combination T1 map and ratio images, generate priors that capture the structures of interest well when projected back into subject space and overlaid onto individual brainstem images. However, the three image output performed better regarding the low silhouette index.

## Data Availability

The data that support the findings of this study are openly available in OpenNeuro https://doi.org/10.18112/OPENNEURO.DS000221.V2 (2017) (LEMON) and Dryad http://doi.org/10.5061/dryad.fb41s (ATAG).
