# Supplementary figures and images for: Mapping internal brainstem structures using MP2RAGE derived T1 weighted and T1 relaxation images at 3 and 7 T
Source: Hum Brain Mapp. 2020 Jan 23;41(8):2173–86. doi: 10.1002/hbm.24938 (PMC7198362; doi:10.1002/hbm.24938)

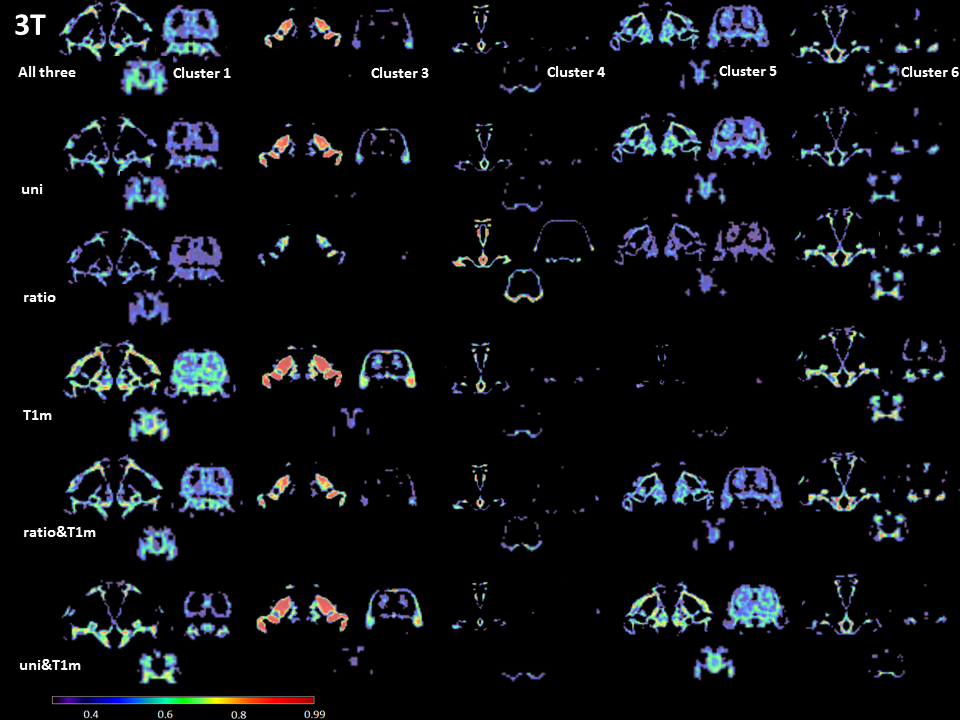

Supplement: Supplementary file 1 — Figure S1 Panels (a) and (b) show the probabilistic segmentation maps or priors generated when using all three images as done in the manuscript or when only using one image type or a combination of two image types as input for the cluster analysis at 3 and 7 T. All prior images were thresholded to show only probabilities ≥0.3. The following qualitative criteria were used to assess the quality of the resulting priors: (1) Presence of clear probability gradients, that is, high probability (>0.9) in the center of the key structures recognized by each cluster and low probabilities (<0.3) at their boundaries. (2) Different structures are separated by the clusters, that is, are represented in one cluster and not distributed between two or more clusters that each show a faint imprint of the structure. (3) The quality of the segmentation of gray matter structures (Clusters 1, 4, and 6) is considered to be more important than that of white matter structures (Clusters 2 and 5). Using these criteria, the following observations can be made. The three image‐based segmentations show good quality at both field strengths.The segmentations based on the UNI image as sole inputs do also perform well at both field strengths but the gray matter clusters are slightly less pronounced than that of the 3 image segmentations, that is, perform worse on criterion 1 than the 3 image approach.The same observations apply to the segmentations obtained with the ratio image as sole input at 3 T and 7 T. The exception is cluster 4 that shows more partial volume voxels (= miss‐classified voxels) in tissue/csf boundary regions then the 3 image versions. In addition to this, the white matter segmentations derived from the ratio image as sole input are less pronounced than in the 3 image version, that is, also perform worse on criterion 1 than the 3 image approach.The results obtained from the T1 maps as sole input differ between the field strengths. At 7 T, gray matter Clusters 1 and 2 are slightly less [file HBM-41-2173-s001.zip › HBM_24938_supplementary_figure_1a.tif]

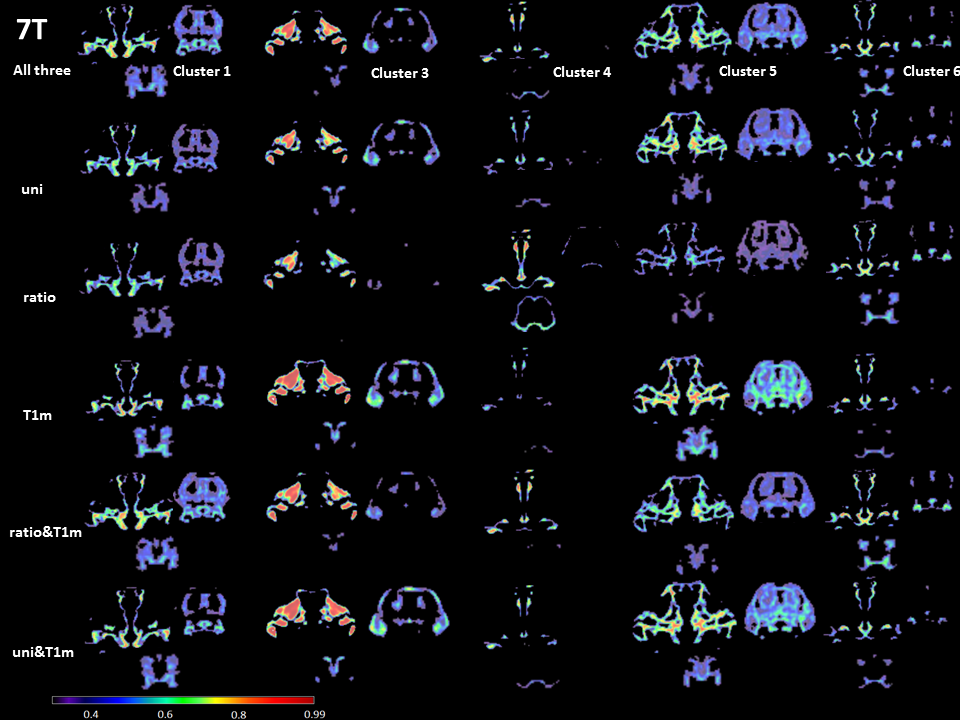

Supplement: Supplementary file 1 — Figure S1 Panels (a) and (b) show the probabilistic segmentation maps or priors generated when using all three images as done in the manuscript or when only using one image type or a combination of two image types as input for the cluster analysis at 3 and 7 T. All prior images were thresholded to show only probabilities ≥0.3. The following qualitative criteria were used to assess the quality of the resulting priors: (1) Presence of clear probability gradients, that is, high probability (>0.9) in the center of the key structures recognized by each cluster and low probabilities (<0.3) at their boundaries. (2) Different structures are separated by the clusters, that is, are represented in one cluster and not distributed between two or more clusters that each show a faint imprint of the structure. (3) The quality of the segmentation of gray matter structures (Clusters 1, 4, and 6) is considered to be more important than that of white matter structures (Clusters 2 and 5). Using these criteria, the following observations can be made. The three image‐based segmentations show good quality at both field strengths.The segmentations based on the UNI image as sole inputs do also perform well at both field strengths but the gray matter clusters are slightly less pronounced than that of the 3 image segmentations, that is, perform worse on criterion 1 than the 3 image approach.The same observations apply to the segmentations obtained with the ratio image as sole input at 3 T and 7 T. The exception is cluster 4 that shows more partial volume voxels (= miss‐classified voxels) in tissue/csf boundary regions then the 3 image versions. In addition to this, the white matter segmentations derived from the ratio image as sole input are less pronounced than in the 3 image version, that is, also perform worse on criterion 1 than the 3 image approach.The results obtained from the T1 maps as sole input differ between the field strengths. At 7 T, gray matter Clusters 1 and 2 are slightly less [file HBM-41-2173-s001.zip › HBM_24938_supplementary_figure_1b.tif]
